# Effect of Intentional Bias on Agency Attribution of Animated Motion: An Event-Related fMRI Study

**DOI:** 10.1371/journal.pone.0049053

**Published:** 2012-11-14

**Authors:** Naoyuki Osaka, Takashi Ikeda, Mariko Osaka

**Affiliations:** 1 Department of Psychology, Graduate School of Letters, Kyoto University, Kyoto, Japan; 2 Department of Psychology, Graduate School of Humanities, Osaka University, Osaka, Japan; University of Regensburg, Germany

## Abstract

Animated movements of simple geometric shapes can readily be interpreted as depicting social events in which animate agents are engaged in intentional activity. However, the brain regions associated with such intention have not been clearly elucidated. In this study, intentional bias was manipulated using shape and pattern animations while measuring associated brain activity using event-related functional magnetic resonance imaging (fMRI). Twenty-five higher-intention involved and twenty-five lower-intention involved animations were presented to participants. Behavioral results showed that the degree of agency attribution of the mental state increased as intentional involvement increased. fMRI results revealed that the posterior superior temporal sulcus (STS), inferior temporal gyrus (ITG), inferior frontal gyrus (IFG), premotor, temporal pole, supramarginal gyrus, and superior parietal lobule (SPL) were activated while participants viewed the high-intention animations. In contrast, occipital, lingual, and middle frontal gyri were activated while the participants viewed the low-intention animations. These findings suggest that as agent attribution increases, the visual brain changes its functional role to the intentional brain and becomes a flexible network for processing information about social interaction.

## Introduction

Recent evidence from cognitive social neuroscience has accelerated our understanding of intricate social brain functions, including processes involving the perception of others and their apparent behavior. However, relatively little research has been conducted to evaluate agency and its role in intentional bias. Moreover, there is limited evidence regarding how the intentional brain can be differentiated from the visual brain. For example, some configural cues such as contingent movement of geometrical patterns trigger an agency or animacy detectors in the brain that can partially explain intentional agents such as other people's minds.

We hypothesize that the specific intentional brain function of estimating others' mental states based on agency attribution is an extended version of the visual brain. This extension involves recruiting higher brain regions found in the temporo-parietal cortices like the superior temporal sulcus (STS) [1]. The social braininvolves consciousness of one's own and others' mental states, intentions, attitudes, beliefs and motives and, therefore, is closely related to the theory of mind (ToM) and intentional agents. The ToM requires the ability to estimate the intentional states of others. Estimating another's state of mind involves modeling the other person's intention, possibly by agency attribution and one's own past experience.

Current social neuroscience studies suggest that the superior temporal sulcus (STS) and medial prefrontal cortex (MPFC) are likely essential components of the social brain region involved in intentional tasks. In order to examine this issue, we developed simple animations that manipulated intentional bias (higher- and lower-intention involved animations) by representing geometrical shapes as opposed to complex verbal or visual tasks.

In their seminal research, Heider and Simmel (1944) [2] and Michotte (1963) [3] used simple moving geometrical patterns as intention-involving agents in a local environment (i.e., a house having walls and a door). In Heider and Simmel's classic experiment, observers were asked to interpret a moving-picture film in which three geometrical figures (i.e., a large triangle (“T”), a small triangle (“t”) and a circle (“c”)) moved in various directions. A rectangle (“house”) with a wall section that opened and closed as a door was also shown. In their original film sequence, the animation was as follows. When the door opened, “t” and “c” moved into the “house.” Then, “T” moved into the “house” and shut the door. Next, “T” and “t” fought and “T” won. Finally, “t” and “c” broke through the door and ran away from the house. This work suggests that moving shapes can simulate the actions of living beings and, therefore, can represent agents performing actions. Accordingly the moving shapes are perceived to have goals and to possess qualities of an intentional mind. Therefore, the moving shapes are likely observed as if they represent the intentional states of others.

In his theory of interpersonal relations, Heider proposed that individuals perceive and create explanations for the behavior of other's, a process he called “attribution” [4]. Researchers have documented that higher-order cognition involving concepts such as causality and agency can be elicited by observing interactions, but not by observing the independent random movements of simple geometrical objects. If animations could possibly evoke mental state attributions based on intention, we propose that attributions of a mental state can be applied to animated objects. If this supposition is true, it would suggest that the neural substrate associated with understanding intentional events would include the same substrate (i.e., the STS) that becomes active when watching an interactive animated object in cooperation with other regions [5]. To date, however, there have been few empirical studies to investigate why and how these attributions are affected by animations containing objects with lower- or higher-intentional involvement.

In mentalization studies in which the ability to estimate another's mind is required, the observer must infer and model the intentions of another person. In this type of paradigm, the observer models the behavior of the other person prospectively by using attributions that are represented as animated dots or cartoons. For example, Baron-Cohen et al. (1994) [6] found a rCB (regional cerebral blood flow) increase in the orbitofrontal cortex of the right hemisphere during the TOM task. Abel, Happe, and Frith, using two triangles moving around the screen in one of three ways (ToM-like, in a goal-directed way, or randomly), compared the attribution of the mental state in autistic children having less TOM than that of normal children, finding that the former used mentalizing (ToM-like) descriptions less often than the latter did [7].

In another study, Schultz et al. presented short animations to participants in which two moving disks appeared to be either interacting or moving independently from each other [8]. Using fMRI, they found that activation in the STS increased in proportion to the degree of correlation between the motion of two disks, and that an increase in correlation increased the amount of interactivity and animacy the observers attributed to the two disks.

Perception of animacy also influences interactive behavior [9]. Recent fMRI studies using non-Heider & Simmel patterns showed that the STS is also activated by simple moving objects whose interactions appear causal or intentional [10] and that the STS is involved in the representation of observed intentional actions [11]. Saxe et al. presented a real movie of a human walking into a room with or without occlusion (e.g., bookcase), finding that the walking figure activated the right posterior STS, which appears to be sensitive to the relationship between the observed motion and local environment [11]. They further hypothesized that the right posterior STS is involved in the representation of observed intentional actions.

In a study using PET, Castelli, Happe and Frith presented participants with a silent, computer-generated animation involving two simple geometric shapes (e.g., triangles) that resembled Heider and Simmel patterns [12]. They found that the STS, MPFC, and temporal regions, including the fusiform gyrus, temporal pole, and occipital gyrus, were activated. The investigators argued that these animations strongly evoked mental state attributions based on intentions and hypothesized that the ability to make inferences about another's mental state evolved from the ability to make inferences about another's apparent behavior. Their findings suggest that controlling the degree of intention from high to low evoked by animations that vary in attribution appears to be critical in this type of research. They had six adult participants observe an animation that involved two moving triangles that manipulated the degree of intention from high to low in three ways: 1) ToM-like, corresponding to high intention; 2) goal-directed, corresponding to intermediate intention; and 3) randomly, corresponding to low-intention intention. These stimuli could therefore be graded from random movements to goal-directed actions, and finally to complex intentional states.

The primary goal of the current work was to evaluate the degree to which intentional bias could result in greater STS activation and less MPFC activation. Similar animations were used such that objects always stayed within the same local region. However, animations differed in terms of their movements. Specifically, some animations were designed to give a graded impression of either intentional-oriented interactions or mechanical-oriented movements [13]. In other words, a primary aim of our study was to describe how the social brain is influenced by animations that evoke high intention relative to less or no intention. We sought to replicate and extend the findings of Castelli et al. [12] using a larger sample and event-related technology, and by grading stimuli based upon random movements, goal-directed actions, and complex intentional states.

## Methods

### Participants

Twelve healthy, right-handed participants (4 males and 8 females; mean age = 25.2) and fifteen separate participants (11 males and 4 females, mean age = 25.8) were recruited for the fMRI experiment and preliminary rating study, respectively. All had normal or corrected-to-normal vision, and were screened for the presence of current or past neurological and psychiatric disorder.

### Ethics Statement

The experiment was conducted in accordance with the guidelines of the ethical committees of the Brain Activity Imaging Center (ATR, Kyoto, Japan) and of Kyoto University. All individuals voluntarily participated in the study and provided their written, informed consent prior to study participation.

### Procedure

**Figure 1:**
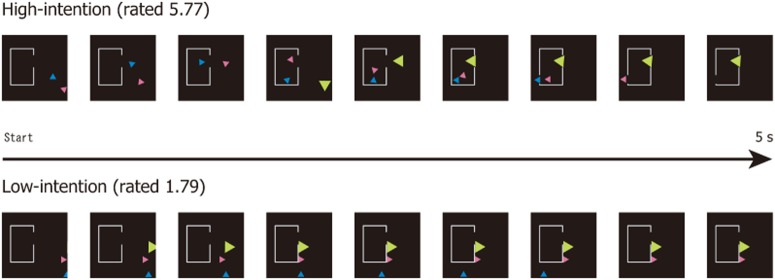
Typical animation strips from high- and low-intention groups, each 5 seconds in length from left to right. Three geometrical objects of different colors (blue, red, and green triangle) move around a black background containing a “house,” which has a gap on its side wall. Preceding the experiment, 2 sets of 25 animation movies each were developed that involve high- and low-intentionality groups. The movies varied in terms of the ratio of degree of attribution of mental states to animated pattern. For example, when the door opened, blue and red move into the “house”. Then, green moves into the “house” and shuts the door. Green and blue fights and green wins. Blue and red broke the door and they ran away from the “house” under the highest intentionality condition (rated 5.77), while figures move in parallel under the lowest intentionality condition (rated 1.79).

The animations used in the study was modeled on that of Heider and Simmel [2]. Figure 1 depicts examples of the five-second animations (moving from left to right) used. Two or three triangles of different colors (blue, pink, and green) moved around on a black background. These triangles corresponded to the “t,” “c,” and “T” stimuli used in the Heider and Simmel animation. Additionally, the animation had a “house” with a gap on its side wall.

**Figure 2:**
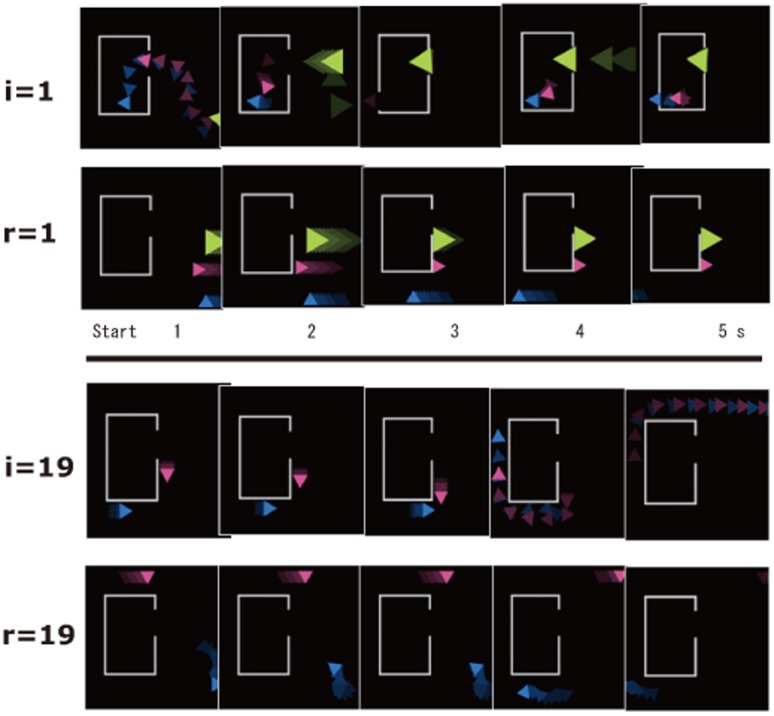
Samples of matched animation sequence from left to right with a 1 s interval between sequences. The upper panel depicts matched pairs (i = 9, r = 9; three triangles) and the lower panel depicts other matched pairs (i = 19, r = 19; two triangles). I = intention; R = random.

The upper panel of Figure 1 shows a high-intention-involved animation (rated 5.77 and corresponding to condition i = 1 in Figure 2; see movies for details). In our preliminary study (see below), one participant reported a ToM-like story corresponding to the high-intention-involved animation as follows: “When the door of the ‘house’ opened, the blue and pink triangles moved in. Then, a green triangle moved in. Green and pink fought and green won. Blue and pink broke out of the ‘house’ and ran away. Based on this script, the two triangles were chased and persecuted by the green triangle and each triangl moved in an interactive way.

The lower panel of Figure 1 depicts a low-intention-involved animation (rated 1.79 and corresponding to condition r = 1 in Figure 2; see movie file in detail). In our preliminary study (see below), a typical response to a story corresponding to one of the low-intention-involved animations as follows: “Triangles moved merely randomly or drifting without interaction”. By varying the motion path of the triangles, 25 different pairs consisting of one high- and one low-intentionality animation were designed for a total of 50 animations. Interactive motion (two triangles chased and persecuted by the third triangle) was varied by the experimente. In order to test the effect of the number of objects, we used three triangles in all but six pair in which the green triangle did not appear. The animations were created and encoded using Adobe Flash CS3 (30 flames per second, 320×320 pixel).

### Preliminary study

In the preliminary behavioral study, 15 participants rated each animation based on an intentionality score. The intentional score was rated using a Likert-type scale of 1 to 7 (1: not at all intentional; 7: highly intentional). Next we selected 25 “high” and 25 “low” intentionality animations. Observers were asked to rate intentionality between the blue object and the other objects based on their mutual actions.

Pairs of high- and low-intention animations were created. Their paths of motion are shown in Figure 2. The highest-intention animation was created in a manner similar to the Heider and Simmel [2] pattern (Figure 1 upper panel, which corresponds to i = 1 motion path in Figure 2). The lowest intention (i.e., random) animation was made by simple drifting (Figure 1 lower panel, which corresponds to r = 1 motion path in Figure 2). We also made a series of different intermediate animation pairs for a total of 25 pairs ranging from (i = 1, r = 1) to (i = 25, r = 25), where i and r indicates intention and random, respectively. Thus, we matched animation to have a similar motion path length and time for all triangles within a pair. Based upon this design, it was expected that participants would judge the triangles in a pair (for example, i = 19, r = 19 shown in Figure 2) to be somehow different from each other in terms of intentionality, while triangles in another pair (for example, i = 1(highest), r = 1(lowest) shown in Figure 1 and 2) would be much different from each other Thus, we created a total of 25 graded steps of stimulus pairs. Of the 25 animations, the mean intentionality score was 5.77 in the “high” group and 1.79 in the “low” group.

A two-way repeated-measures ANOVA (intention×animation number) revealed a significant main effect for intentionality [F(1,14) = 768.9, *p*<.001] and stimulus number [F(24,336) = 4.82, *p*<.001]. We also found significant interaction between intentionality and stimulus number [F(24,336) = 6.35, *p*<.001]. Multiple comparisons using Turkey's HSD revealed significant differences between high- and low-rated scores. Thus, we confirmed that the higher-rated group was significantly more sensitive to intention than the lower-rated group. T-tests comparisons between the number of objects (2 to 3 objects) found no differences in terms of intentionality. Based on these preliminary findings, we adopted all the stimulus objects tested for later experiments.

### fMRI session

No participants who participated in the preliminary study participated in the fMRI study. In an fMRI session, an animation was presented one second after a beep tone and an evaluation screen appeared which asked the participant to rate the level of intentionality from one (high) to four (low). Participants made ratings by choosing from two sets of four buttons (one set for each hand). One trial took 17 s, resulting in a total of length of 14 min 30 s for each session. For the first session, fifty moving patterns were presented in random order to participants in a counter-balanced manner. Twenty-five patterns were presented to the high group and to the low group, respectively. In the second session, up-down reversed patterns from the first session were presented. The presentation of normal and up-down reversed patterns was counter-balanced for each participant. In the preliminary study, we confirmed that participants could easily decide a response after reading the agent's intention 3 s after presentation. Therefore, the fMRI scan began 3 s after the animation presentation.

Animations were back-projected onto a screen viewed through an angled mirror. The size of each animation was 11.5°×11.5°. In one session, participants observed 50 animations presented in random order. The length of each trial was 17 seconds.

### fMRI data acquisition

Whole brain images were acquired on a 1.5-T whole-body magnetic resonance imaging scanner (Shimadzu-Marconi Magnex Eclipse, Kyoto, Japan). Head motion was minimized with a forehead strap. Functional MRI was performed with a gradient echo-planer imaging (TR = 3000 ms, TE = 49 ms, flip angle = 90°, 5 mm slice thickness, FOV = 192 mm×192 mm, and pixel matrix 64×64). After the collection of functional images, T1-weighted images (154 slices with no gap) using a conventional spin echo pulse sequence (TR = 12 ms, TE = 5 ms, flip angle = 8°, FOV = 220 mm×220 mm, and pixel matrix 256×256) were collected for anatomical co-registration with the functional images.

After image reconstruction, functional images were analyzed using SPM2 (Wellcome Department of Imaging Neuroscience, London, UK). Six initial images were discarded from the analysis to eliminate the non-equilibrium effects of magnetization. All functional images were corrected for between-slice timing differences in image acquisition and realigned to correct for head movement, which was less than 1 mm within runs. The functional images were normalized and spatially smoothed with an isotropic Gaussian filter (6 mm full-width at half-maximum). Low-frequency noise was removed by high-pass filtering (time constant = 128 s). We conducted the analysis using an event-related design. An onset of an event according to the data analysis occurred three seconds after an animation started based on the results of the preliminary study.

Data were modeled by convolving the vector of expected neural activity with the canonical hemodynamic response function (HRF) included in SPM2 and modulated by ratings of intentionality (4-point scales: high for 4 and low for 1). Single-participant *t*-contrast images were then entered into second-level analysis using a random effects model for all participants. The levels of statistical significance for these analyses were set to *p*<0.001 (uncorrected).

## Results

**Figure 3:**
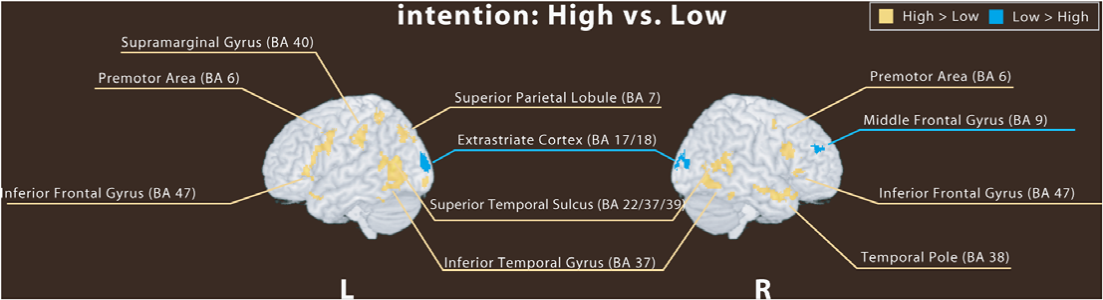
Brain activation regions for high→low-intention corresponding to the social brain (i.e., yellow area) and areas for low→high-intention corresponding to the perceptual brain (i.e., blue area). Event-related fMRI results showed that main activation areas occurred in three regions while participants observed low-intention animations: extrastriate cortices including calcarine sulcus and lingual gyrus (BA 17,18), and right middle frontal gyrus (BA9). During high-intention animations, activation of more widespread regions was observed, including: bilateral inferior frontal gyrus (BA47:IFG), premotor (BA6:PM), superior temporal sulcus (BA22/37/39: STS), inferior temporal gyrus(ITG), left supramarginal gyrus (SMG), left superior parietal lobule (BA 7: SPL), and right temporal pole (BA38:TP).

Two contrasts were specified per single-participant analysis: 1) Low versus High and 2) High versus Low. Low-intention involves activations under participant's button press 1 (highest) and 2 (higher) and high-intention involves that of button press 3 (lowest) and 4 (lower). As shown in Figure 3 and Table 1, fMRI revealed activation of three main areas when participants observed 25 low-intention-involved animations (low>high): the left middle occipital areas including the calcarine sulcus/cuneus (BA17,18), the right lingual gyrus (BA18), and the right prefrontal gyrus in the middle prefrontal cortex (BA9). However, when participants observed 25 high-intention-involved animations and intentional bias was increased (high>low), the activated areas extended to include the bilateral posterior STS sulcus (BA22/37/39), the right temporal pole (BA38), the bilateral inferior frontal gyrus (BA47:IFG), the premotor (BA6), the inferior temporal gyrus (ITG), the left supramarginal gyrus, and the left superior parietal lobule (SPL). We did not find any activation in the MPFC (Table 1).

**Table 1:** Brain region of activation for each contrast.

		BA	*x*	*y*	*z*	*Z*
Region of activation						
**Low>High**						
Middle frontal gyrus	R	9	30	46	20	4.11
Calcarine sulcus/cuneus	L	17/18	−10	−92	16	4.48
Lingual gyrus	R	18	12	−70	−2	3.93
**High>Low**						
Inferior frontal gyrus	L	47	−51	25	2	4.97
	R	47	50	20	19	3.83
Premotor area	L	6	−53	5	20	4.42
	R	6	44	4	50	3.87
Superior temporal sulcus	L	37/39	−55	−62	10	4.83
Superior temporal sulcus	R	22/37	48	−42	11	4.81
Temporal pole	R	38	44	7	−21	4.63
Inferior temporal gyrus	R	37	48	−68	2	4.48
Supramarginal gyrus	L	40	−57	−27	35	4.28
Superior parietal lobule	L	7	−32	−41	67	4.03
Parahippocampal gyrus	L	36	−26	−43	−8	3.96

Note: Uncorrected *p*<0.001, BA, Brodmann area:

L, Left: R, Right.

## Discussion

In this study, we sought to investigate the differential contributions of the areas involved in visual and intentional cognitive processes. Participants conducted tasks that required them to make social interpretations by looking at moving objects that were presented as low- or high-intentionally biased animations. By varying the stimuli, we varied the extent to which intentional cognitive processing was required, which facilitated the analysis of intentional and perceptual influences on various brain regions.

Based upon event-related fMRI data, our results revealed activation of several visual areas including the calcarine sulcus/cuneus and the lingual gyrus (BA17, 18), which is near the fusiform gyru when the visual brain operated in a mechanical low-intention-involved context. The middle frontal gyrus is thought to maintain visual attention to groups of moving objects [13]. In contrast, the fusiform gyrus is believed to play a general role in the representation of visual stimuli that signify intent, independent of the visual form [14]. Our finding of activation in the lingual gyrus, which is near the fusiform gyrus corroborates with a previous study [14].

As shown in Table 1, when the brain processes high-intention-involved interactive animations, activation in the posterior STS involving part of the supramarginal area increased. It has been demonstrated that the STS becomes activated while viewing animated geometrical figures portraying social interactions [5], [13], [14] and when evaluating the intentions of others. Using fMRI, Gobbini et al. [14] reported that social animations activated an extensive portion of the STS including areas in the posterior STS as well as the inferior parietal lobule.

In an earlier PET study, Castelli et al. [12] presented animations that featured two characters (a large red triangle and a small blue triangle) moving on a framed white background similar to Heider and Simmel's pattern [2]. The investigators presented each participant with three types of animation: 1) ToM (two triangles bluffing one another); 2) goal-directed (two triangles dancing together); and 3) random (two triangles merely drifting). These animations were displayed for approximately 40 s over the course of 12 scans and divided into two consecutive counterbalanced blocks consisting of cued and uncued animations. These animations were designed to evoke mentalizing and elicited activity in the STS relative to a random motion condition. The design of the current study improved that done by Castelli et al. in two ways. First, intentional biases were manipulated continuously from highest to lowest by 25 matched pairs selected from 50 animations using ratings from the participants in a preliminary study. Second, an event-related design was introduced to avoid prior knowledge by using a shorter presentation duration (5 s). Based on our results, it is likely that intentional bias may be controlled more by the STS than by the MPFC, particularly when brain responses to high-intention-involved animations are compared with responses to low-intention-involved animations.

The STS has been hypothesized to be closely connected to the perception of biological motion. Studies using transcranial magnetic stimulation [15] and magnetoencephalography [16] have shown that the simulation of human walking induced by moving dots selectively activates a brain area on the ventral bank in the occipital extent of the STS and the right temporo-parietal junction. Furthermore, such animations may be similar to the Heider and Simmel [2] paradigm. We show here that tasks tapping mentalization and agency attribution activated the same brain regions in the STS and temporo-parietal cortices including the supramarginal gyrus, inferior temporal gyrus, the temporal pole, and the SPL. One explanation for why we did not find activation in the MPFC is that we used an event-related design to avoid expectancy with a much shorter presentation time than the 30 s previously reported [12]. Expectancy cueing and longer presentation time could also yield possible contingent activations in the MPFC in addition to controlling intentional bias in the STS. It is highly possible, therefore, that higher-intention-involved animations, such as the fight between the blue and green triangle used in the current study, was perceived by the observer as though he/she was participating in the action against an antagonis. Indeed, humans may possibly detect intentions in shapes, even when those shapes change their motion to face another object [9].

Overall, we assumed that activation in the premotor cortex invoked a mirror system when a human acts and when the person observes the same action performed by anothe [17]. This system may be important for understanding the actions of other people, and that of the geometrical shapes in our animations. Some researchers also speculate that mirror systems may simulate observed actions, thus contributing to ToM skills [18], [19]. In the premotor area, a functional mirror system estimating others' intentions may contribute to activation of the IFG [20]. In the current study, significant increases of activation in the IFG were observed only when the animations were actively viewed with intention. Therefore, it is possible that the IFG monitors intentional thoughts in the STS. In contrast, activity in visual areas, including the lingual gyrus, which is near the fusiform gyrus, was only found in conditions requiring less intentional involvement and passive viewing.

With close interconnections to the STS, the IFG and the temporal pole provide internally-represented self and other's mental states. Rather than the MPFC per se, it is the ventral side of the IFG, close to the orbitofrontal PFC and temporal pole, along with temporo-parietal-junction areas including the posterior STS and supramarginal gyrus [11]) that are possible critical components for the representation of another's mental state. Saxe et al. [11] examined whether activation of the posterior STS, similar to the perception of intentionality, depends particularly on the contingency between an agent's motion and the environment by introducing short and long occlusions of a walking person's animation strip. They showed that right posterior STS activation occurred following the long occlusion (i.e., when a person remained hidden for a few seconds before re-emerging). In the current study, we found activation in the same region; namely, the bilateral posterior STS, using simple geometric animations depicting high-intention-involved action. The present study suggests that the posterior STS is involved in constructing an abstract visual description of another agent's intentional actions, without engagement of the MPFC. Based on the present results, it is possible that incoming animated information is decoded perceptually and integrated with contextual interpretation; the constituent product of these two processes can be understood either in terms of perceptual- or intention-involved behaviors.

In their examination of the neural correlates of mentalization, Vogeley et al. (2001) [21] used fMRI to investigate common and differential neural mechanisms underlying ToM and the self during the presentation of a verbal story, finding that a ToM task led to increased neural activity in the temporal pole, whereas the self-task led to increased neural activity in the right temporo-parietal junction involving the STS. Interestingly, our data corroborate theirs regarding the neural correlates of ToM despite the large differences in the methods employed. The ability to model another intentional mind using an animated patter could be an evolutionary innovation in the human social brain that developed from the perceptual brain. Further investigations are necessary in order to clarify this issue.

## Conclusion

To summarize, we investigated how the visual brain transitions to the social brain using event-related fMRI in the present study. Animations consisted of moving patterns evoking various mental states of attribution based on intentions. Among 25 pairs of animations, each participant rated the higher- and lower-intention animation according to their attribution of agency (i.e., internal or external). Results showed that activations of the posterior STS, ITG, IFG, premotor, temporal pole, supramarginal gyrus, and SPL occurred under high-intention–involved animations, whereas occipital, lingual, and middle frontal gyri were activated under lower-intention-involved animations.

Findings of the present study suggest that as intentional stance increased, the portion of the social brain involving the representation of an agent's intentional actions became more activated. Thus, developing the capacity to model another's mind could be an evolutionary innovation in the human social brain that developed from the perceptual brain. Previous studies have implicated regions activated by higher intention in self-monitoring in the perception of biological motion and in the attribution of mental states, and regions activated by lower-intention in simple perceptual processing. In the present study, we report how the visual brain shifts to the social brain in an agency attribution experiment. We suggest that as agent attribution increases, the visual brain changes to the intention-assuming social brain and therefore possesses a flexible network for processing information about social interactions based on agency attribution.

## Supporting Information

Movie S1 **An example movie under high-intention condition.**
(AVI)

Movie S2 **An example movie under low-intention condition.**
(AVI)

Movie S3 **An example movie under intermediate high-intention condition.**
(AVI)

Movie S4 **An example movie under intermediate low-intention condition.**
(AVI)
